# Chest CT findings of COVID-19-infected patients, are there differences between pediatric and adult patients? A systematic review

**DOI:** 10.1186/s43055-020-00261-8

**Published:** 2020-08-04

**Authors:** Javid Azadbakht, Hamed Haghi-Aminjan, Bagher Farhood

**Affiliations:** 1 Department of Radiology, Faculty of Medicine, Kashan University of Medical Sciences, Kashan, Iran; 2 Pharmaceutical Sciences Research Center, Ardabil University of Medical Sciences, Ardabil, Iran; 3 Department of Medical Physics and Radiology, Faculty of Paramedical Sciences, Kashan University of Medical Sciences, Kashan, Iran

**Keywords:** 2019 novel coronavirus, COVID-19, Chest CT, Thoracic CT, Lung, Pulmonary, Pediatric, Adult

## Abstract

**Background:**

Purpose of this study was to deliver a report of chest CT findings of COVID-19-infected pediatric and adult patients and to make an age-based comparison. A systematic search was conducted in accordance with PRISMA guidelines to identify relevant studies in the electronic databases of PubMed, Scopus, ProQuest, ScienceDirect, and Web of Sciences from January 1, 2020 to March 27, 2020 using search terms in the titles and abstracts. Based on our inclusion and exclusion criteria, 762 articles were screened. Finally, 15 eligible articles which had adequate data on chest CT findings of COVID-19-infected patients were enrolled in this systematic review.

**Results:**

In pediatric patients (15 years old or younger), peripheral distribution was found in 100% of cases, ground glass opacities (GGO) in 55.2%, bilateral involvement in 50%, halo sign in 50%, unilateral involvement in 30%, consolidation in 22.2%, crazy paving pattern in 20%, nodular opacities in 15%, pleural effusion in 4.2%, lymphadenopathy in none, and normal imaging in 20.8% of cases. On the other hand, in adult patients, bilateral involvement was reported in 76.8%, GGO in 68.4%, peripheral distribution in 62.2%, mixed GGO and consolidation in 48.7%, consolidation in 33.7%, crazy paving pattern in 27.7%, mixed central and peripheral distribution in 25.0%, unilateral involvement in 15.2%, nodular opacities in 9.2%, pleural effusion in 5.5%, central distribution of lesions in 5.4%, lymphadenopathy in 2.4%, and normal imaging in 9.8% of cases.

**Conclusion:**

According to the findings of this systematic review, children infected with COVID-19 can present with normal or atypical findings (nodular opacities/unilateral involvement) in chest imaging more frequently than adult patients. Therefore, more caution should be taken to avoid misdiagnosis or missed diagnosis in infected children. Besides, clinical and laboratory findings need to be considered more decision-making for pediatric patients with normal or atypical chest CT scan but high suspicion of COVID-19.

## Background

Coronaviruses are enveloped single-stranded RNA viruses which can rapidly mutate and recombinate and have basic reproductive number of about 2.2 [1]. Six coronavirus species can result in human disease [2]. Four species—229E, OC43, NL63, and HKU1—are prevalent which in immunocompetent individuals typically induce common cold symptoms [2]. The two other species—SARS-CoV and MERS-CoV—are originally zoonotic and occasionally have been connected to fatal illness [3]. Due to high prevalence and wide distribution of coronaviruses, considerable genetic diversity and frequent genome recombination, and increasing activities which bring human and animal together, novel coronaviruses are possible to periodically infect humans because of frequent cross-species infections and occasional virus spillover [3, 4].

WHO (the World Health Organization) received data on some patients with unknown cause respiratory disease from Wuhan City, China, on December 31, 2019, with clinical presentations similar to those of viral pneumonia including symptoms of as fever, cough, and dyspnea. Also, patients often showed pulmonary opacities at chest imaging [5]. Bronchoalveolar lavage fluid sample analysis and electron microscopy showed coronavirus as causative infectant, a virus with a crown like morphology at electron microscopy resulted from viral spike peplomers coming out from the viral envelope [6]. This newly found strain has been temporarily named the 2019 novel coronavirus (2019-nCoV or COVID-19). The virus is highly contagious and can be transmitted from human to human by an infected person or an asymptomatic carrier and is capable of spreading rapidly between cities. Respiratory droplets are the main route of transmission, but close contact and transmission through digestive tract can play a role also [7]. Most of the cases are mild in clinical term, but the elderly or those with comorbidities are more likely to experience severe form of infection [8].

Humankind is generally susceptible to COVID-19 and no vaccination or definite treatment is at hand yet. Early detection and effective control of transmission pathways (i.e., isolation of suspected cases, disinfection, etc.) are still the most effective procedures to stop the COVID-19 outbreak. However, because of large number of clinically suspected COVID-19 patients, laboratory detection tests take a lot of time hindering early treatment and may not be feasible for all suspected patients owing to the low number of available COVID-19 test kits [9]. Moreover, due to the limitation of sampling materials in the early stage of the infection, the positive rate is relatively low [10]. These challenges increase spread chance by free movement of highly suspected cases who are not yet identified and quarantined.

Imaging plays a crucial role in the diagnosis and follow-up of novel coronavirus-infected pneumonia (NCIP) patients. CT is regarded as preferred imaging modality in clinically suspected cases and is valuable to monitor imaging changes during treatment. Therefore, CT has been considered as an effective diagnostic tool for clinically suspected COVID-19 [9]. It potentially can spot people with negative reverse transcription–polymerase chain reaction (RT-PCR) but yet highly suspicious of NCIP regarding clinical conditions [10, 11]. CT scan findings may also provide information about the disease severity [12–15]. Furthermore, considering similarities in imaging patterns of most viral pneumonia, the CT features reported in many recent studies might be helpful to discriminate virus from other pathogens causing pneumonia and to triage patients.

Some studies have reported CT features of patients infected with COVID-19. However, chest CT findings of pediatric patients with COVID-19 infection may be different from adults. In this study, we aimed to review literature systematically to find differences in reported CT features in COVID-19-infected patients in pediatric and adult age group, considering major CT role in screening and triage of COVID-19 disease where there is no serological testing available or results are not returning in due time.

## Methods

### Study protocol

The present study was done according to the guidelines of Preferred Reporting Items for a Systematic Review and Meta-analysis (PRISMA) [16].

### Search strategy

A systematic search was performed in electronic databases including PubMed, Scopus, ProQuest, ScienceDirect, and Web of Sciences from January 1, 2020 to March 27, 2020. The selected keywords were in accordance with our study purpose, “Diagnostic Imaging” OR “Diagnostic X-Ray” OR “Diagnostic X-Ray Radiology” OR “Medical Imaging” OR “X-Ray Computed Tomography” OR “CT” OR “X-Ray Computer Assisted Tomography” OR “CT X-Ray” OR “X-Ray CAT Scan” OR “X-Ray Computerized Tomography” AND “COVID-19” OR “2019-nCoV” OR “nCoV” OR “coronavirus” OR “SARS-CoV-2” OR “Wuhan coronavirus” OR “Novel coronavirus” OR “2019 novel coronavirus” OR “coronavirus disease 2019 virus” OR “COVID-19 virus” OR “2019-nCoV infection” OR “coronavirus disease-19.”

### Eligibility criteria

After collecting the articles, their title and abstract were evaluated and then, all eligible studies related to our aims and with sufficient data were included in the present study. For exclusion criteria, we excluded (1) posters, (2) review articles, (3) case reports, (4) letters to the editor, (5) the articles with not comprehensive data, and (6) not available article.

### Data collection

After the first screening of the articles in accordance with the above-mentioned keywords in their title and abstract, the second screening was performed for finding the relevant full-text articles based on the inclusion and exclusion criteria and finally, the eligible articles were selected. All the screening process was performed independently by two reviewers (BF and HHA).

## Results

### Literature search and screening

**Fig. 1:**
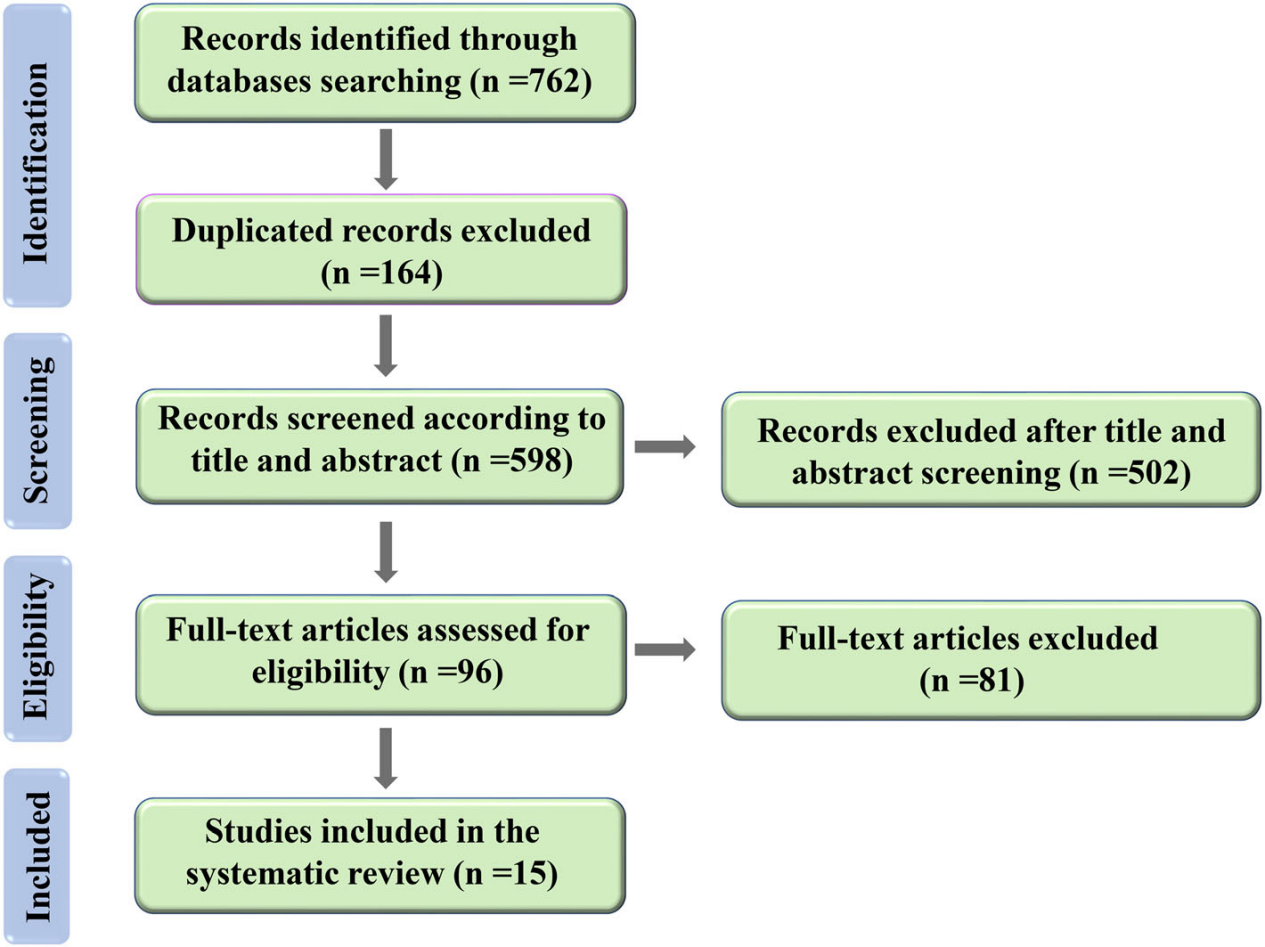
PRISMA flowchart used for the study selection

Seven hundred sixty-two articles were found by a comprehensive searching on the above-mentioned electronic databases and 164 duplicated articles were removed. Five hundred ninety-eight articles were included for the first screening, 502 articles were omitted, and 96 articles were screened in their full texts. In the final step, 15 articles were eligible to be included in present study which had adequate data on chest CT findings of COVID-19-infected patients, were in English language, and contained RT-PCR-confirmed cases. The procedure of the literature searching and screening method are shown in Fig. 1.

### Data extraction

The data of each article including author name, number of patients, and chest CT findings of pediatric and adult patients infected with COVID-19 were extracted and listed in Table 1.

**Table 1:** The CT findings of pediatric and adult patients infected with COVID-19

Author name	No. of cases (P:A)	Chest CT findings of patients																											
		GGO		Consolidation		GGO and consolidation		Halo sign		Nodular opacities		Crazy paving		Lymphadenopathy		Pleural effusion		Unilateral involvement		Bilateral involvement		Central distribution		Peripheral distribution		Central and peripheral distribution		Normal radiology	
		P	A	P	A	P	A	P	A	P	A	P	A	P	A	P	A	P	A	P	A	P	A	P	A	P	A	P	A
Li et al. [17]	5 (5:0)	60	NA	0	NA	0	NA	NA	NA	NA	NA	NA	NA	NA	NA	NA	NA	NA	NA	NA	NA	NA	NA	NA	NA	NA	NA	NA	NA
Xia et al. [18]	20 (20:0)	60	NA	NA	NA	NA	NA	50	NA	15	NA	20	NA	NA	NA	0	NA	30	NA	50	NA	0	NA	100	NA	NA	NA	20	NA
Liu et al. [19]	59 (4:55)	25	80	50	42	NA	61	NA	NA	NA	NA	NA	41	0	0	25	23	NA	25	NA	68	NA	1	NA	99	NA	NA	25	6
Xie et al. [11]	5 (0:5)	NA	60	NA	0	NA	40	NA	NA	NA	NA	NA	NA	NA	NA	NA	NA	NA	0	NA	100	NA	0	NA	100	NA	0	NA	NA
Yoon et al. [20]	9 (0:9)	NA	45	NA	5	NA	50	NA	NA	NA	10	NA	10	NA	NA	NA	NA	NA	NA	NA	NA	NA	20	NA	60	NA	20	NA	NA
Xu et al. [21]	90 (0:90)	NA	72	NA	13	NA	NA	NA	NA	NA	NA	NA	12	NA	1	NA	4	NA	NA	NA	59	NA	NA	NA	51	NA	NA	NA	23
Zhao et al. [22]	80 (0:80)	NA	71	NA	27.8	NA	NA	NA	NA	NA	NA	NA	NA	NA	1.3	NA	1.3	NA	5	NA	95	NA	NA	NA	NA	NA	NA	NA	NA
Shi et al. [23]	81 (0:81)	NA	65	NA	NA	NA	NA	NA	NA	NA	6	NA	10	NA	6	NA	5	NA	NA	NA	79	NA	NA	NA	54	NA	NA	NA	NA
Wang et al. [24]	114 (0:114)	NA	27.3	NA	27.3	NA	45.4	NA	NA	NA	NA	NA	NA	NA	NA	NA	0.9	NA	NA	NA	85.4	NA	NA	NA	NA	NA	NA	NA	2.6
Liu et al. [25]	137 (0:137)	NA	NA	NA	NA	NA	NA	NA	NA	NA	NA	NA	NA	NA	NA	NA	NA	NA	NA	NA	84.7	NA	NA	NA	NA	NA	NA	NA	NA
Wang et al. [26]	138 (0:138)	NA	NA	NA	NA	NA	NA	NA	NA	NA	NA	NA	NA	NA	NA	NA	NA	NA	NA	NA	100	NA	NA	NA	NA	NA	NA	NA	NA
Chung et al. [6]	21 (0:21)	NA	57	NA	0	NA	29	NA	NA	NA	0	NA	19	NA	0	NA	0	NA	10	NA	76	NA	NA	NA	33	NA	NA	NA	14
Yuan et al. [27]	27 (0:27)	NA	67	NA	19	NA	30	NA	NA	NA	7	NA	NA	NA	0	NA	4	NA	15	NA	85	NA	0	NA	26	NA	74	NA	0
Huang et al. [12]	41 (0:41)	NA	NA	NA	NA	NA	NA	NA	NA	NA	NA	NA	NA	NA	NA	NA	NA	NA	NA	NA	98	NA	NA	NA	NA	NA	NA	NA	NA
Song et al. [15]	51 (0:51)	NA	77	NA	55	NA	59	NA	NA	NA	NA	NA	75	NA	6	NA	8	NA	14	NA	86	NA	10	NA	86	NA	2	NA	0
Totally	878 (29:849)	55.2 (3/16/29)	68.8 (10/367/533)	22.2 (2/2/9)	23.4 (9/107/457)	0 (1/0/5)	48.2 (8/136/282)	50 (1/10/20)	NA	15 (1/3/20)	5.8 (4/8/138)	20 (1/4/20)	27.7 (6/85/307)	0 (1/0/4)	2.4 (7/10/405)	4.2 (2/1/24)	3.5 (8/18/519)	30 (1/6/20)	11.7 (6/28/239)	50 (1/10/20)	82.1 (12/991/1207)	0 (1/0/20)	5.4 (5/8/147)	100 (1/20/20)	62.2 (8/211/339)	NA	25 (4/23/92)	20.8 (2/5/24)	6.7 (6/30/449)

*NA* not applicable, *GGO* ground glass opacity, *P* pediatric, *A* adult

All of numbers are percentages except for numbers in parenthesis in last row which are presentative of (studies engaged/cases positive for a specific finding/all of cases) respectively. In study [11], 100% of lesions was subpleural, which implies presence of peripheral distribution in 100% of cases. Also in this study, authors reported crazy paving as fine mesh shadow. In study [20], GGO prevalence (35%) was added to crazy paving prevalence (10%) because of same nature in crazy paving except for interlobular septal thickening which occurs in it (In this article, these two were reported separately.). This study was not considered in last row calculations because imaging findings were not per case, but per lesion. In study [19], 4 children and 55 adult patients (pregnant and non-pregnant) were enrolled. For adult age group, average of 3% reported in this article (for non-pregnant adults group, laboratory-confirmed pregnant women group, and clinically diagnosed pregnant women group) is presented (for decimal numbers, they were rounded down). In this article, GGO with reticulation was considered as crazy paving pattern. Regarding very small sample volume in pediatric age group, numbers in this study are affected by small sample size bias. In study [24], single or multiple lesions in one lung was reported to be 9.1% and 5.5% respectively, so 85.4% of cases had bilateral involvement

### Chest CT findings of pediatric patients with COVID-19 infection

In three studies [17–19] which reported CT features in pediatric patients infected with COVID-19 (aged 15 or younger), one also included adult patients.

Ground glass opacities (GGO) frequency reported as 60% [17], 60% [18], and 25% [19]. Consolidation frequency reported to be 0% [17] and 50% [19], but only one study reported mixed GGO and consolidation as 0% [17]. Pleural effusion in children reported as 0% [18] and 25% [19] and normal imaging was seen in 20% [18] and 25% [19] of cases.

Pooling data from all studies, GGO was found in 55.2% of pediatric patients, consolidation in 22.2%, normal imaging in 20.8%, and pleural effusion in 4.2% of cases. Lymphadenopathy frequency was reported in a study by Liu et al. [19] as 0%. Xia et al. reported halo sign in 50% of pediatric age group, nodular opacities in 15%, crazy paving in 20%, and peripheral distribution (subpleural lesions) in 100% of cases [18].

### Chest CT findings of adult patients with COVID-19 infection

GGO, consolidation, and mixed density frequency were in a wide range in literature (27.3 to 80% [6, 10, 15, 19–24, 27], 0 to 55% [6, 10, 15, 19–22, 24, 27], and 29 to 61% [6, 10, 15, 19, 20, 24, 27] of cases, respectively). Also, considerable diversity in reported frequencies of nodular opacities and crazy paving pattern were noted (0 to 10% [6, 20, 23, 27] and 10 to 75% [6, 15, 19–21, 23], respectively). Lymphadenopathy (0 to 6% [6, 15, 19, 21–23, 27]), pleural effusion (0 to 23% [6, 15, 19, 21–24, 27]), unilateral involvement (0 to 25% [6, 10, 15, 19, 22, 27]), central distribution (0 to 20% [6, 10, 15, 19, 22, 27]), and normal imaging (0 to 23% [6, 15, 19, 21, 22, 27]) were less frequent features, as opposed to bilateral involvement (59 to 100% [6, 10, 12, 15, 19, 21–27]) and peripheral distribution (26 to 100% [6, 10, 15, 19–21, 23, 27]) which was higher than central distribution frequency in corresponding study in all of cases, which were more encountered.

Pooling data from all studies, bilateral involvement was reported in 76.8% of adult patients, GGO in 68.4%, peripheral distribution in 62.2%, mixed GGO and consolidation in 48.7%, consolidation in 33.7%, crazy paving pattern in 27.7%, mixed central and peripheral distribution in 25.0%, unilateral involvement in 15.2%, nodular opacities in 9.2%, pleural effusion in 5.5%, central distribution of lesions in 5.4%, and lymphadenopathy in 2.4% of patients. A total of 9.8% of adult patients had normal imaging.

## Discussion

Normal imaging was reported more frequently in infected children comparing to adults (20.8% versus 6.7%). Most frequent CT feature for pediatric patients was peripheral distribution and with decreasing order of frequency next features were GGO, bilateral involvement, unilateral involvement, consolidation, crazy paving pattern, nodular opacities, pleural effusion, central distribution, lymphadenopathy, and mixed consolidation and GGO (three latter features were not seen in any of investigated patients). Most frequent CT feature for adult patients was bilateral involvement and with decreasing order of frequency next features were GGO, peripheral involvement, mixed GGO and consolidation, consolidation, crazy paving pattern, mixed central and peripheral distribution, unilateral involvement, nodular opacities, pleural effusion, central distribution, and lymphadenopathy. Hereby, CT findings reported in the studies on both pediatric and adult cases enlisted above are discussed and compared (this list will not include CT features not reported in pediatric patient studies.).

### GGO and consolidation

GGO is a hazy gray (slightly increased density) area without bronchovascular obscuration, causing by partial air displacement resulting from two possible mechanism, alveoli partial filling or interstitial thickening [28]. Postmortem biopsy in a COVID-19 patient showed pulmonary edema and hyaline membrane formation in both lungs [29], which can be pathologic explain of GGO [30]. Moreover, as mentioned above, there was diversity in results from different studies regarding lesion density frequency. This could be explained by symptom onset to CT scan interval. Patients with longer time interval between symptom onset and CT scan will have more consolidative lesions [15]. Lesion density gradually increased to consolidation in up to 2 weeks after disease onset in a study by Pan et al. [31], which is in consistency with a study by Shi et al. which showed that GGO can progress to or co-existed with consolidations within 1–3 weeks [23].

Consolidation reflects alveolar air occupied by fluids, cells, or tissues and manifest as density higher than GGO, high enough for bronchovascular obscuration [28]. Cellular fibromyxoid exudates in alveoli may play a role in consolidative lesions in COVID-19 [29]. For consolidation and mixed density, data available for children are from two studies with small sample size [8, 19] and further studies are needed to compare these CT findings in adults and children.

But generally, GGO, consolidation, and mixed GGO and consolidation all seem to occur with less prevalence in pediatric patients.

### Halo sign

**Fig. 2:**
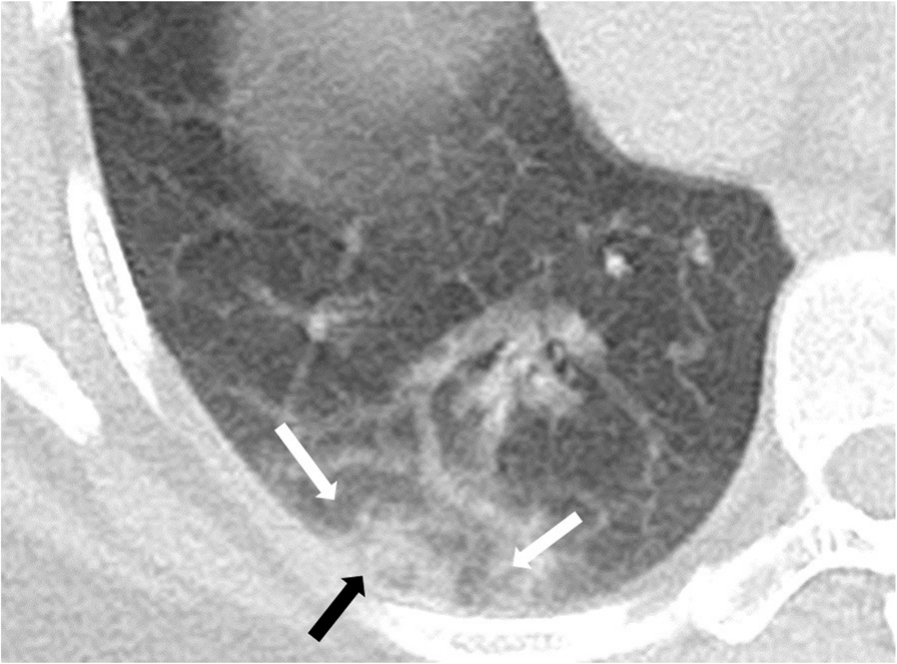
Halo sign: ill-defined focus of consolidation (black arrow) is surrounded by a rim of GGO (black arrow). Various entities can give a halo sign; hence, it is rather a nonspecific sign

Halo sign is consolidative nodule or masse with peripheral ground glass opacity [28] (Fig. 2). It can be associated with viral infections and organizing pneumonia [32]. Halo sign frequency was reported as 50% in pediatric patients [18] and is claimed to be uncommon in adults [33] and just mentioned in a case report [29] and a case series with 3 cases [34].

### Nodular opacities

**Fig. 3:**
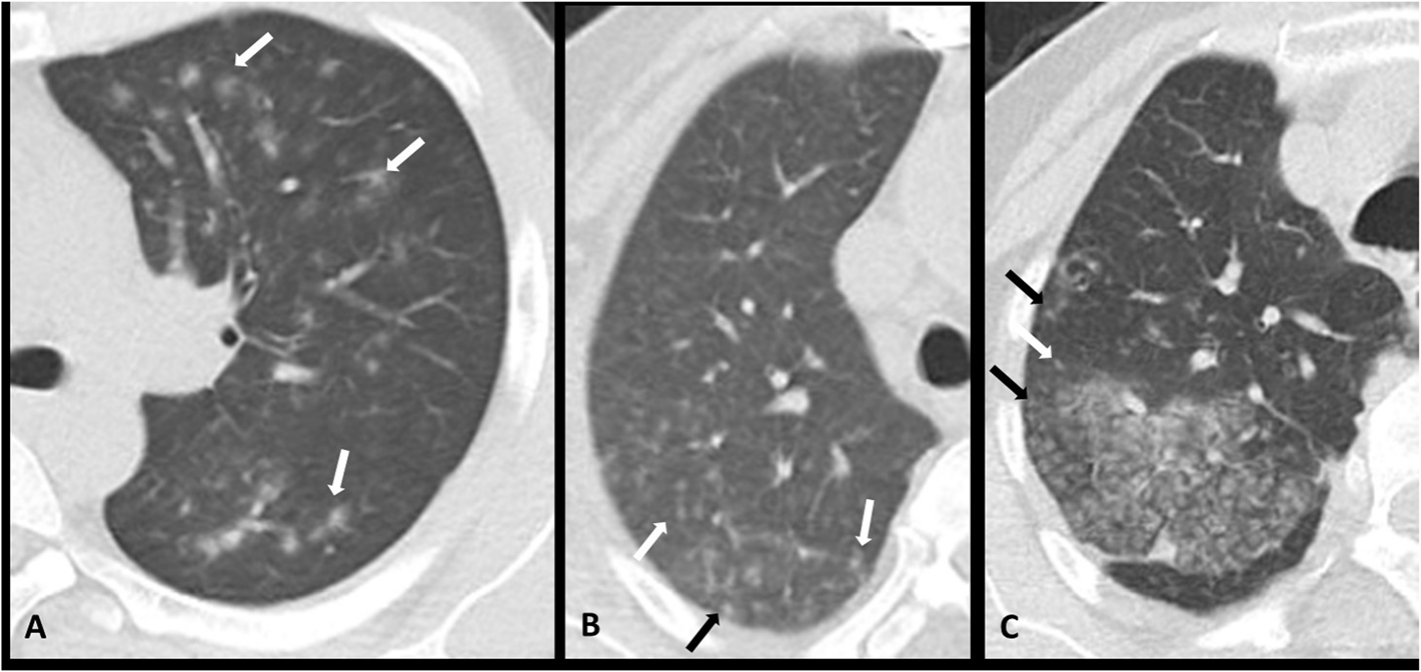
Nodular opacities: small and round, ill-defined nodules of soft tissue density (some indicated with arrows) in 3 different patients with RT-PCR-confirmed COVID-19

Nodule is defined as well or poor demarcated rounded or irregular opacity with diameter less than 3 cm [28] (Fig. 3) which is frequently associated with viral pneumonia [35]. Its frequency in infected children was slightly higher than adult patients (15% versus 9.2%). Again, only one study has reported nodular opacity frequency in children [18].

### Septal thickening and crazy paving

Reticular opacities (interlobular and intralobular septal thickening) might be at least partly due to interstitial lymphocyte infiltration [29]. Reticular opacity pattern frequency increases in later stage of COVID-19 patients [23]. To our knowledge, no paper has investigated reticular pattern in children, but in adults it has been reported in some studies as 33% in Shi et al. study [23] and 67% in Song et al. study [15].

**Fig. 4:**
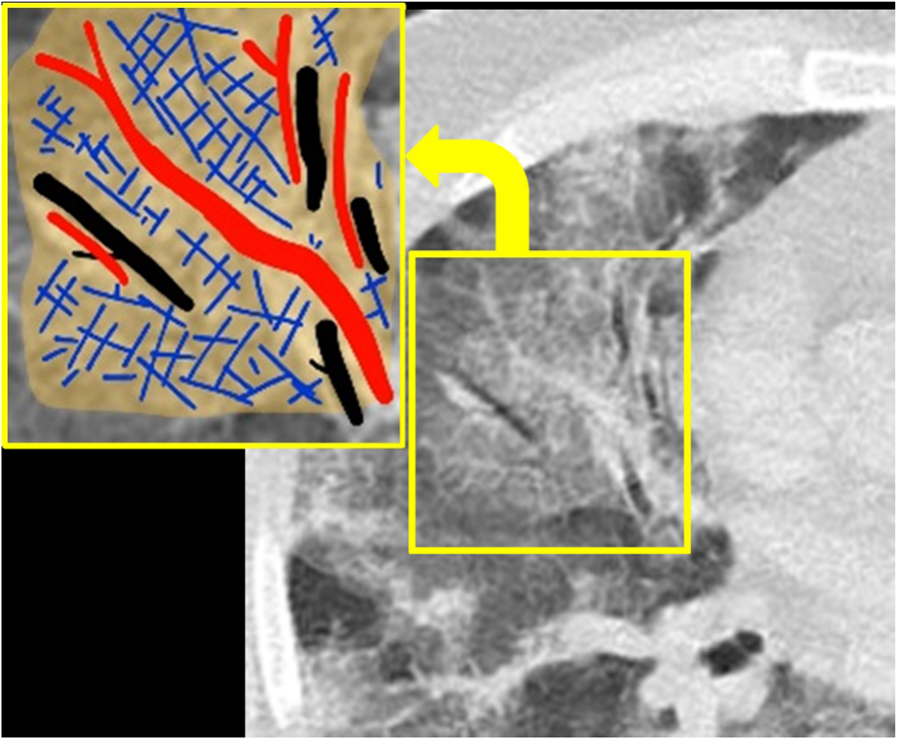
Crazy paving: thickened interlobular septa (hued blue in enhanced insert) on a GGO background (highlighted in yellow) creates similar appearance to a pavement made up of disorganized paving stones. Colored in black and red are air bronchogram and pulmonary vessels

If reticular opacity superimposes on GGO background, it looks like paving stones hence so called crazy paving pattern [28] (Fig. 4). Pathologic explain for this can be alveolar edema and interstitial inflammation due to acute lung injury [11, 36]. It can herald infection entering progressive or peak stage [31]. Crazy paving prevalence is reported as 20% in children [18] which is slightly less frequent than that of adult patients (27.7%). More studies with larger sample size are needed for children to provide a more presentative frequency. Also, studies on adults which take time interval between symptom onset and CT scan into consideration will troubleshoot diversities in reported prevalence.

### Lymphadenopathy

Short axis diameter of 1 cm for mediastinal nodes is considered as thresholds discriminating enlarged nodes (lymphadenopathy based on size criterion) from normal size nodes [28]. Lymphadenopathy is one of significant risk factors of severe/critical COVID-19 pneumonia [37]. If lymphadenopathy coincides with pleural effusion and numerous lung nodules, it may suggest bacterial superinfection [13]. Lymphadenopathy is rare in both children and adults, but only one study on pediatric patients reported it with 4 cases, and none of them had lymphadenopathy [19].

### Pleural effusion

Pleural effusion is less frequent comparing to pleural thickening and occurred in 5% of adult cases in Shi et al. study [23]. Pleural effusion can be an indicator of poor prognosis in NCIP [23]. An autopsy report by Xi Liu et al. showed pleural thickening and extensive adhesion in an adult patient with COVID-19 which is what that is seen as pleural thickening with or without visible pleural effusion in CT scan [38]. Pleural effusion was rare in both children and adults (4.2% and 3.5% respectively).

### Bilateral involvement

Unlike SARS and MERS, where initial chest imaging abnormalities are more frequently unilateral, COVID-19 is more likely to involve both lungs on initial imaging [39]. In a systematic review by Salehi et al., bilateral involvement in 919 cases was reported as 87.5% [33]. Although bilateral involvement was more frequent in both adults and pediatric patients, but unilateral involvement was more frequent in children comparing to adults (30% versus 11.7%). Lieu et al. reported bilateral involvement in 20% (1/5) of pediatric patients, which again emphasizes on trend toward unilaterality of lesions in pediatric patients [40] {Liu, 2020 #2}.

### Axial distribution

**Fig. 5:**
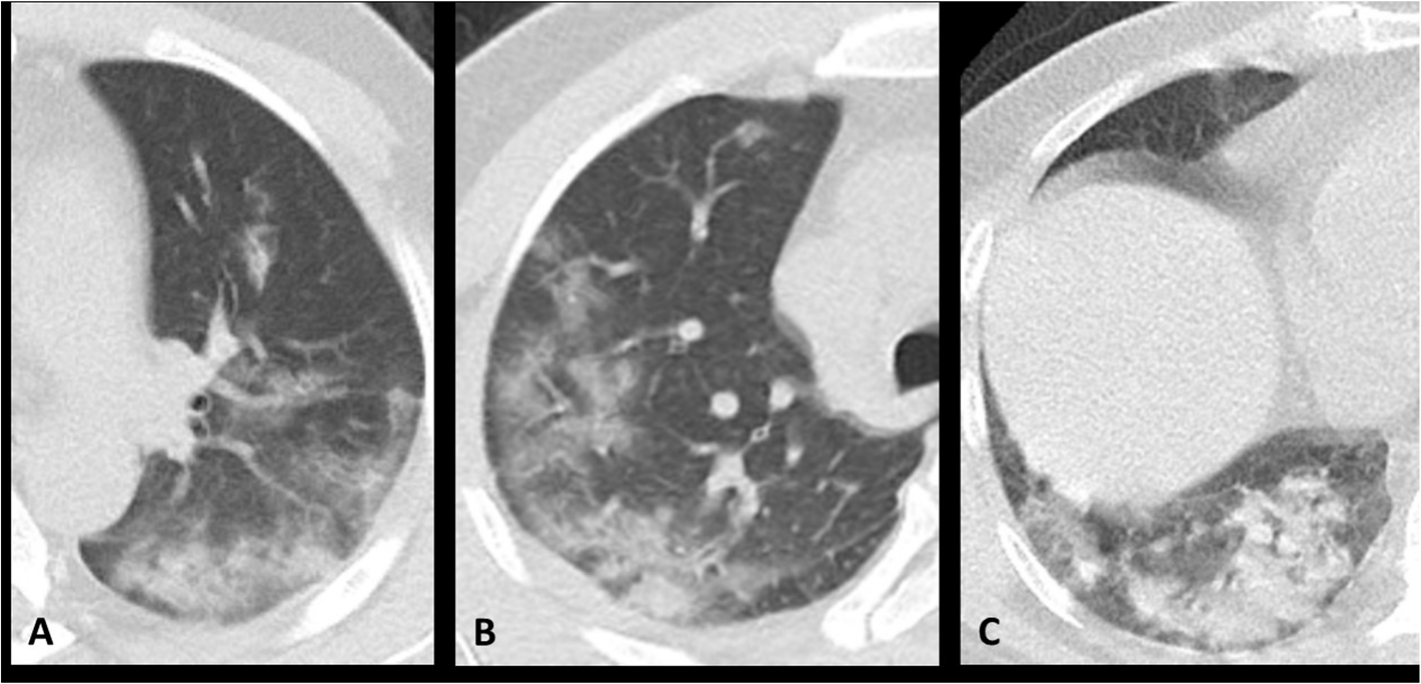
Peripheral distribution: COVID-19-related pneumonia is presented as peripheral lesions in most of cases. Peripheral distribution is even more encountered in pediatric patients

In several studies, it has been stated that peripheral (subpleural) distribution is typical for NCIP (Fig. 5). Salehi et al. reported 76% peripheral distribution in 121 patients (enrolled in 12 studies) [33]. In children, peripheral distribution frequency was 100% (yet more prevalent than infected adults), although this difference can be under influence of relatively small sample size in only study which reported it [18].

Vascular enlargement, reticular opacities, air-bubble sign (which was described in one study by Heng et al. [30]) or lucencies and cavities in opacified areas, air bronchogram, reverse halo (atoll) sign, craniocaudal distribution (upper/middle or lower zone involvement), and one to five lung lobes’ involvement frequency (and frequency of two or more lobes’ involvement) are features investigated in several adult studies, but no pediatric patients’ study has been done to assess this finding prevalence yet; hence, as an interesting issue in future studies, assessment of the above-mentioned CT features in pediatric patients is suggested. This study has its limitations to be noted. First, number and sample size of pediatric patient studies was small and this can influence on imaging features’ prevalence; hence, we suggest more studies with larger sample sizes to be conducted on pediatric age group with NCIP for one can compare them with infected adults’ chest imaging features. Second, time gap between symptom onset and CT can influence on prevalence of findings mentioned above, regarding any CT feature time course. Categorizing CT findings in predetermined time line stages would provide more reliable frequencies and comparisons.

## Conclusion

In conclusion, children infected with COVID-19 can present with normal or atypical findings (nodular opacities/unilateral involvement) in chest imaging more than adult patients; therefore, more caution should be taken to avoid misdiagnosis or missed diagnosis in infected children, and clinical and laboratory findings need to be considered more decision-making for pediatric cases with normal or atypical chest CT scan but highly suspicious of COVID-19.

## Data Availability

Not applicable

## Abbreviations

CT: Computed tomography
GGO: Ground glass opacities
2019-nCoV: 2019 novel coronavirus
NCIP: Novel coronavirus-infected pneumonia
RT-PCR: Reverse transcription–polymerase chain reaction
PRISMA: Preferred Reporting Items for a Systematic Review and Meta-analysis
